# Sex differences in phenotypic modulation of microglia by early-life physical stress in a rat model of chronic primary low back pain

**DOI:** 10.1007/s00424-025-03125-0

**Published:** 2025-11-08

**Authors:** Deepika Singhal, Jonathan R. Husk, Wolfgang Greffrath, Rolf-Detlef Treede

**Affiliations:** 1 Department of Neurophysiology, Medical Faculty Mannheim, Mannheim Centre for Translational Neuroscience, Heidelberg University, Mannheim, Germany; 2 Department of Psychiatry and Psychotherapy, Medical Faculty Mannheim, Central Institute for Mental Health, Heidelberg University, Mannheim, Germany

**Keywords:** Chronic primary low back pain, Latent sensitisation, Nociceptive priming, Neural inflammation, Musculoskeletal pain

## Abstract

Chronic primary low back pain (cpLBP) is a leading contributor to years lived with disability. Early-life stress is a major risk factor predisposing to cpLBP later in life upon minor injuries. We investigated sex differences in the involvement of microglia in the pathophysiology of early-life stress effects on pain responses to a secondary stimulus in adulthood. During adolescence, male and female Wistar Han rats underwent repeated restraint stress for 12 consecutive days, while controls were handled. In adulthood, acute LBP was induced by NGF or saline injections into the lumbar multifidus muscle. Subsequently, the animals were sacrificed and perfused for spinal cord extraction. A total of 3516 microglia cells were classified into three functional states (surveillant, primed, activated) using partition around medoids clustering and UMAP dimensionality reduction methods for eight 3-dimensional morphological features obtained from MATLAB 3DMorph. Across all conditions, the proportion of surveillant microglia was significantly higher in females than in males (*p* < 0.0001, *d* = 1.85), while males had more primed (*p* < 0.0001, *d* = 1.56) and activated (*p* < 0.01, *d* = 1.87) microglia. Priming by stress led to an increase in activated microglia after NGF injection (*p* < 0.05, *d* = 0.63), more distinct in males (*p* < 0.05, *d* = 0.82) than in females (*p* > 0.05, *d* = 0.43). Additive effect of stress and NGF caused a shift towards primed state in males (*p* > 0.05, *d* = 0.62), but not in females. In conclusion, stress was confirmed to play a critical role in priming microglia and predisposing to cpLBP. Sex differences previously shown for neuropathic pain were found to be also relevant in this more frequent musculoskeletal pain condition.

## Introduction

Chronic primary low back pain (cpLBP) is a leading contributor to years lived with disability globally [18]. In contrast to chronic secondary low back pain, which is often linked to alterations in bones and joints, it is related to dysfunctions in muscles and fasciae. Recent evidence confirms that the source of pain is more likely of fascial than muscular origin [10]. A wide range of treatment modalities have been tried, mostly with small effect sizes as recently reviewed in a WHO guideline on its management [3].

We have established a rodent model of cpLBP by administering two low-dose injections of nerve growth factor (NGF) into the multifidus muscle [21, 22, 44, 45, 51, 53, 54, 57, 69] [58]. When injected in humans, these doses induce hypersensitivity to painful test stimuli but not spontaneous pain [16]. In rats, the first NGF injection induces only transient muscle hypersensitivity, but hypersensitivity after the second injection is long-lasting and accompanied by sensitisation of dorsal horn neurons (DHNs) to muscle inputs. This phenomenon is known as the double-hit model of NGF sensitisation, wherein the first conditioning injection leaves a state of latent sensitisation (memory trace), which allows the second injection to have a prolonged efficacy, inducing manifest sensitisation. Microglial signalling plays a major role in initiation of this latent sensitisation [51, 69]. The pro-inflammatory cytokines released by activated microglia enhance excitatory synaptic transmission in the spinal cord, hence contributing to pain hypersensitivity [8, 15, 46]. Morphological changes in microglia, e.g. caused by chronic stress [9], can serve as markers to distinguish between activated and non-activated microglia.

Adverse childhood experiences (ACEs) significantly increase the risk of developing chronic primary pain in adulthood, with affected individuals facing a 1.45 to 1.95 times higher likelihood of experiencing chronic pain [12]. To study ACEs and their effects on a cellular and molecular level, various animal models can be utilised, and previous studies have revealed the pivotal role of ACEs in modulating pain perception and intensity [20, 35, 42, 60]. Different stressors can influence pain pathways by disrupting systemic hormonal and immune signalling, and inducing neural remodelling in the central nervous system (CNS) [32]. Neuroimmune interactions, particularly those involving proinflammatory cytokines and glial cells, play a critical role in the development and progression of pain and its comorbidities [25, 47], and chronic stress has been found to induce increased and long-lasting microglial priming [50]. It has been reported that mouse models of chronic stress exhibit enhanced neuroinflammatory signalling within the spinal cord, potentially contributing to hyperalgesia and allodynia [1, 48].

In this study, we hypothesize that adolescent rats show sex-specific differences in neuro-immune responses to stress, which differentially prime spinal microglial cells that lead to a hyper-responsive pain-processing state. For this purpose, we subjected adolescent rats of both sexes to repeated restraint stress (RS) [53] and challenged their nociceptive system in adulthood by two injections of NGF or saline. We assessed priming by stress, priming by a preceding NGF injection, and the interaction of stress and NGF. To study sex differences in the pathophysiology of early-life stress effects on the pain response to a secondary stimulus in adulthood, we assessed microglia activation in superficial and deep laminae of the spinal cord dorsal horn in both male and female rats.

## Methodology

All experiments were conducted in accordance with the ARRIVE 2.0 guidelines, ensuring rigorous study design, ethical compliance, and transparent reporting (Table 1).

**Table 1:** ARRIVE Guidelines: A checklist of 10 essential items for animal studies

**Fig. 1:**
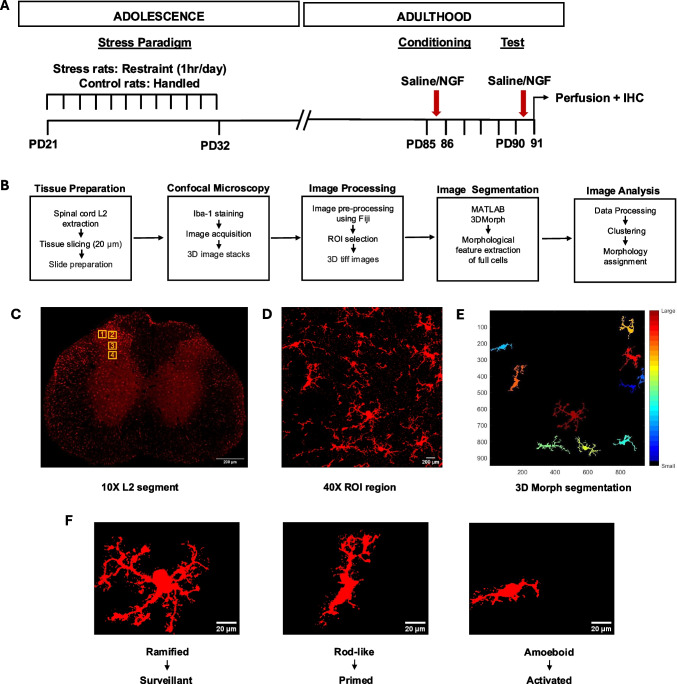
Experimental design for evaluating the activation of microglial cells after early life stress in a model of myofascial low back pain in rats. **A** Experimental Timeline: The animals were divided into four groups: two stress and control groups each. During adolescence, from postnatal day (PD) 21 to PD32, the stress groups were exposed to repeated stress for 12 days using a narrow restrainer while the control groups were handled. In adulthood, on PD85 and PD90, the animals received injections of NGF or saline (as detailed in the methods). The animals were sacrificed on PD91. **B** Procedure Post-Perfusion: The spinal cord was removed and frozen on dry ice after transcardial perfusion. The L2 region of the spinal cord was sectioned into 20 μm slices using a cryostat. The slices were stained with Iba-1 antibodies, and 3D images were acquired for further analysis using a confocal SP8 microscope, including image segmentation and image processing. **C** Iba-1 Stained Image Overview: A 10X magnification image showing the dorsal horn region, with four 256 × 256 μm regions of interest (ROIs) selected for further analysis. **D** 40X Magnification of ROI 1: A closer view of one of the selected ROIs at 40X magnification. **E** Image Segmentation: The 3D Morph microglial segmentation (York et al., 2018) protocol was used to identify and segment whole microglial cells within the ROI and determine the features of each segmented cell. **F** Classification of microglia states: Microglial cells are classified into three major morphological types: Ramified, Rod-like and Amoeboid considered to correspond to the functional states (see Methods Section 2.8)

1. **Study design**
a) Experimental study and parallel group design. (Methods, Sect. “Methodology”) b) The experimental unit is each animal
2. **Sample size**
a) Total of 16 female and 16 male Wistar rats were used (Details in Sect. “Experimental animals”). Sample size and experimental procedure approved by “Regierungspräsidium”, the regional board, Karlsruhe, Germany b) The sample size was based on the previous studies with similar design (Singaravelu et. al., 2022)
3. **Inclusion and exclusion criteria**
a) Four of six best tissue extracted were used for stainings. The tissues with lesions were excluded b) N has been mentioned in Sect. “Experimental animals”*.* in methods
4. **Randomisation**
a) No randomisation was used b) Identical protocol with identical Iba1 + antibodies were used from the previous study (Singaravelu et al., 2022) c) Fully automated cell classification was used for all treatments to minimize the investigator’s bias
5. **Blinding/masking**
The experimenter (D.S.) was blinded to the assignment of the animals into treatment groups. The unblinding code was held by J.R.H., who did not disclose this information to D.S
6. **Outcome measures**
a) 3D acquisition of the Iba-1 + microglia cells in the L2 section of the spinal cord. Automated morphology analysis of the microglia in three states: surveillant, primed, and activated b) This was not a confirmative study. No primary outcomes were defined
7. **Statistical methods**
Pre-processing of data: Cluster analysis Data analysis: Two-way ANOVA, independent t-test. Cohen’s *d*. (Methods Sect. “Image Processing”)
8. **Experimental animals**
a) Adolescent male and female rats (Wistar Han outbred), initial body weight 50 g. Terminal body weight 200–225 g (females) and 385–415 g (males) b) The animals were also monitored for grooming behaviour and abnormal posture
9. **Experimental procedures**
Experimental procedure: stress paradigm described in Sect. “Repeated Restraint Stress”, nerve growth factor or saline injections in Sect. “Rat model of myofascial low back pain”, Treatment groups in Sect. 2.4, Perfusion and tissue preparation in Sect. 2.5. The timeline is highlighted in Fig. 1. The rationale has been mentioned in Introduction (Sect. “Introduction”)
10. **Results**
Data are given as means ± SE of the proportion of the microglia in each state. The details are mentioned in Results (Sect. “Results”)

### Experimental animals

The experiments were performed in compliance with German law on the protection of animals under the permission number QZ 35–9185.81/G-7/19. The study used 16 female Wistar-Han rats. To determine sex differences, the raw confocal images of 16 male Wistar-Han rats from a previous study [53] were reanalysed using a 3D automated protocol developed as part of the present study. The behavioural data (evoked pain) for these animals have been published [55] briefly: Stressed and control animals were tested with mechanical stimuli on PD34 and PD85 (before injections) and post-injections. Adolescent stress resulted in widespread hypersensitivity to mechanical stimulation of muscles and skin in males lasting into adulthood (manifest sensitisation), while females exhibited transient hypersensitivity in adolescence and enhanced responsiveness to injections in adulthood (latent sensitisation).

Animals were obtained from ENVIGO in the Netherlands at postnatal day (PD) 21. Upon arrival, they were housed in groups of four in standard Makrolon cages measuring 55 cm × 35 cm × 20 cm. They were provided ad libitum access to food and water and maintained under a standard 12-h light/dark cycle. Following a brief acclimatisation period of approximately 2 h in their home cages, animals were transferred to the experimental procedure room and allowed an additional 1-h acclimatisation period. All experiments were conducted during the animal’s inactive phase. Half of the animals were randomly selected for the stress paradigms (two per cage). Each cage consisted of two stressed and two control animals. Throughout the study, animals were consistently handled by the same experimenter (DS). The experimenter was blinded throughout the study.

### Repeated restraint stress

Repeated restraint stress (RS) was the stress paradigm used to mimic physical trauma in early adolescence in female rats using the identical paradigm as previously in male rats [53]. Rats randomized for RS were kept inside a narrow cylindrical restrainer (inner length 15 cm; inner height 4 cm) for 1 h daily for 12 consecutive days (Fig. 1). The animals were stressed from PD 21 to PD 32. Control animals were only handled. On PD34, stressed males and females were more sensitive to mechanical stimulation than unstressed controls. On PD85, hypersensitivity was still present in stressed males, whereas stressed females no longer differed from unstressed controls [55]. Body weight was measured every day before beginning the stress paradigm.

### Rat model of myofascial low back pain

For the establishment of the myofascial low back pain model in adulthood, the animals were injected with saline (vehicle) or NGF in the left multifidus muscle on PD 85 and PD 90 [22]. NGF, a human recombinant diluted to 0.8 µM (Calbiochem, MERCK, Germany), was dissolved in phosphate buffered saline (PBS: pH of the NGF solution 7.2–7.3) and 0.9% isotonic saline was used for the injections. Fifty microliters (NGF/saline) was injected into the left multifidus muscle, located 3 mm lateral to the spinous process at vertebral level L5. This concentration of NGF did not cause immediate ongoing pain but induced hyperalgesia [16] when injected into the muscles of humans. Saline injections served as the control, with no observed signs of muscle inflammation. This model has already been reported in several previous studies [21, 22, 51, 53, 54, 69], especially for studying mechanical hyperalgesia of the low back muscle.

### Treatment groups

The NGF injections and the restraint stress paradigm during adolescence were used for interventions. In the study, we used stress paradigms to mimic childhood adversities, and NGF was used as a mild nociceptive input in adulthood. Each group consisted of *n* = 6 rats and was treated as follows:

- Stress + Saline + NGF (SSN): This is the main experimental group used to study if stress could cause latent sensitisation of DHNs. Hence, the animals were stressed in adolescence, and saline was injected on PD 85, followed by NGF on PD 90 (as the nociceptive input in adulthood).
- Control + Saline + NGF (CSN): This is the control group for the effects of NGF without preceding priming by restraint stress. Non-stressed animals are administered NGF only on PD 90.
- Stress + Saline + Saline (SSS): This is the control group for the effects of stress without NGF. Animals experienced stress during adolescence, and two saline injections were administered at PD 85 and PD 90, respectively.
- Control + NGF + NGF (CNN): This is the positive control group where non-stressed animals are injected with two NGF injections consecutively, one for induction of latent sensitisation the second for induction of manifest sensitisation [22].

The following comparisons were analysed according to our hypotheses:

- SSN vs. CSN: activation by NGF with and without previous stress (priming by stress)
- CNN vs. CSN: activation by 2nd NGF with and without previous 1 st NGF (priming by 1 st NGF)
- SSN vs. SSS: synergy between stress and NGF

### Perfusion and tissue preparation

The animals were sacrificed on PD 91 with an overdose of thiopental sodium i.p. (Trapanal, INRESA GmbH, Germany). The animals were transcardially perfused with 4% paraformaldehyde (PFA) in 0.1 M PBS. After perfusion, a laminectomy was performed, and the spinal cord was extracted. The injections were administered at the L5 segment of the spinal cord, while the primary afferent input from the low back multifidus muscle is received at the L2 spinal segment. [58]. Therefore, the L2 segment of the spinal cord was cut and stored in 10% sucrose solution in 0.1 M PBS at 4 °C. The next day, the tissue was transferred to the high osmotic solution of 30% sucrose, also prepared in 0.1 M PBS at 4 °C before freezing, which served as a cryoprotectant, dehydrated the tissue, and prevented the formation of ice crystal artefacts in the frozen tissue sections. The sections were rapidly frozen on dry ice using Tissue-Tek®, an optimal cutting temperature compound (OCT). The tissue sections were cut using a cryostat (Cryostat NX70, Thermo Fisher Scientific Inc., USA). Each 20 µm section was carefully mounted on a clean glass slide. Three tissue sections from four animals in each group were randomly selected for immunohistochemistry.

### Immunohistochemistry

#### Immunofluorescence labelling

The morphology of microglial cells was visualised by staining the microglia-specific calcium-binding protein ionised calcium-binding adapter molecule 1 (Iba-1). The sections were first incubated in 10% Roti-block solution (Carl Roth, Germany) at room temperature for 1 h, followed by primary rabbit anti-Iba-1 polyclonal antibody (1:1000; ABCAM, UK) at room temperature for 16 h or overnight. The next day, the tissue sections were washed thrice for 5 min each using 1X PBS. Later, the sections were incubated in a secondary antibody, Cy3-conjugated goat anti-rabbit IgG antibody (1:500; Jackson Immunoresearch, USA) at room temperature in the dark for 4 h. The sections were again washed thrice in 1X PBS for 5 min each. All the slides were stained with DAPI (1:1000; Thermo Fischer Scientific Inc., USA) for 15 min and were mounted with Roti mounting medium (Carl Roth, Germany). Slides for male animals had been prepared in a previous study using the same protocol and the same antibodies [53].

#### Image acquisition

The images were obtained using the confocal laser-scanning microscope (LEICA TCS SP8 AOBS, Wetzlar, Germany). The fluorescence of the Cy3-conjugated secondary antibody was detected by the 561 nm DPSS laser (Leica Microsystems, Germany). For analysis of single-cell morphology, three-dimensional images of the ipsilateral dorsal horn over the 20 µm z-axis with a step size of 1 µm were acquired using the 40X objective lens. This step was done for slides from female and male animals within the same imaging session.

### Image processing

After acquisition of images in a z-stack, four regions of interest (ROIs) were selected for each image. Each ROI was saved as 256 µm × 256 µm in 3D TIFF images with Fiji. These 3D images were used to segment the microglial cells using the MATLAB-based programme 3DMorph [68]. It is a semi-automated process to retrieve maximum information about individual microglia cells from overlapping 3D clusters. A total of 96 ROIs were run through the 3DMorph MATLAB script and the corresponding features for each cell were extracted. R Studio v 4.3.0 (2023.9.1.494) was further used to combine the data files for each animal, which were used for the classification of the microglial cells. This procedure was also applied to histological slides of the previous study in male rats that had only been analysed using an operator-dependent manual classification (Quote ref 46).

A total of 3516 whole cells were segmented with specific features. Eight features were extracted for morphological classification of the cells [68]:

(A) Cell territorial volume (µm^3^): It is determined by enclosing all extreme points of the microglia cell within a polygon.
(B) Cell volume (µm^3^): It is calculated by converting the number of voxels in each cell into real-world units using a specified scaling factor.
(C) Ramification index: It is the ratio of cell territorial volume to the cell volume. When a cell body occupies only a small fraction of the space reached by its branches, this ratio is high.
(D) Number of endpoints: It is determined as the microglia cell pixels attached to only one other such pixel in a 3D skeletonised cell image.
(E) Number of branchpoints: It is determined as points of intersection between primary, secondary, tertiary, or quaternary branches.
(F) Average branch length (µm): It is calculated by tracing all endpoints to the centroid and averaging the measured lengths.
(G) Max branch length (µm): It is determined by the longest measured distance between an endpoint to the cell’s centroid.
(H) Min branch length (µm): It is determined by the shortest measured distance between an endpoint and the cell’s centroid.

#### Data preprocessing

The feature data set was scaled before the cluster analysis by subtracting the mean and dividing by the standard deviation. Scaling is the process of standardizing or normalizing variables to ensure that all the features contribute equally to the clustering process. Since clustering relies on distance metrics (Euclidean distance was used here), unscaled data with large magnitude differences can dominate the clustering results. In this study, “Standardization (Z-score normalization)” was used; transformed data have a mean of 0 and a standard deviation of 1.

#### Correlation matrix

The correlation was calculated to identify the strength and direction of a linear relationship between pairs of variables. The correlation matrix was selected because the features have different units. Due to the Z-score normalization, the covariance and correlation matrices are numerically equal. To reduce redundancy in the feature space and overfitting, one of the pairs of very highly correlated features (r > 0.9) was to be omitted from further analyses. The feature with the highest average absolute correlation across all other features was also to be removed [43].

#### Clusteranalysis

A distributive cluster analysis was used for clustering the cells that separate data sets for maximal similarity within clusters and dissimilarity between clusters in a multidimensional space. For distributive cluster analyses, the number of clusters has to be predetermined. An “elbow plot” is often used in clustering analysis to determine the optimal number of clusters (k) [61]. This plot analyses the within-cluster sum of squares (WCSS), which quantifies the variance of each cluster. As the number of clusters (k) increases, WCSS typically decreases due to improved data partitioning. However, after reaching a certain point, the reduction in WCSS becomes less significant, indicating that the “elbow point” corresponds to the optimal k. Beyond this point, increasing the number of clusters results in minimal gains in compactness while adding unnecessary complexity to the model.

**Fig. 2:**
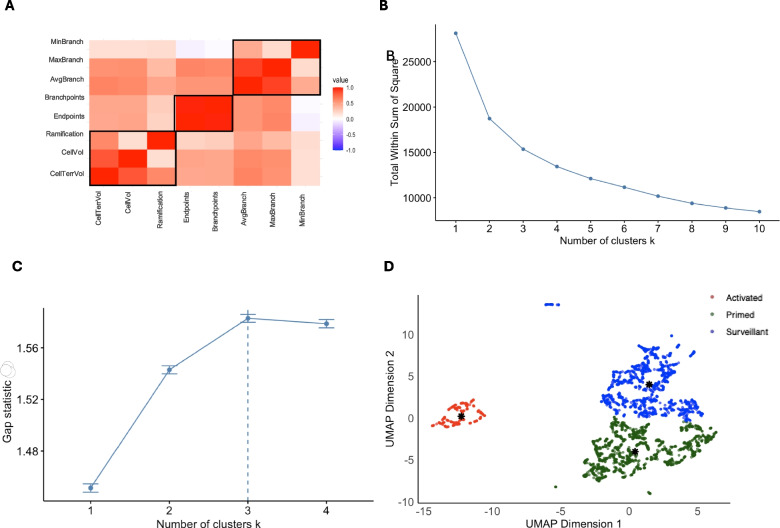
Clustering Analysis. Eight features were acquired from the segmentation process and used further for the analysis to determine the morphology types of the microglia cells. **A** Correlation Matrix: It measures the strength and direction of the linear relationship between 2 variables. One of the highly correlated variables (number of endpoints), r > 0.9, was excluded from the further analysis. **B** Elbow Plot: This method is used to determine the optimal number of clusters (k) by identifying where the decrease in within-cluster variance begins to level off. Here, the bend tends to be between k = 2 and k = 4. **C** Gap Statistics: This is the method used to determine the optimal number of clusters (k) by comparing the within-cluster variance of the actual data to that of a randomly generated dataset. The gap statistic resulted in k = 3, as the highest value signifies more distinct clustering structure. **D** UMAP and cluster visualisation: Uniform Manifold Approximation and Projection (UMAP), a non-linear dimensionality reduction technique to visualise and cluster high-dimensional data. The clustering method called Partitioning Around Medoids (PAM) was used as it is a more robust method than k-means clustering, better suited for datasets with outliers and non-spherical clusters. The clustering divided the data into 3 clusters. The black asterisk (*) represents each cluster’s actual data point as the medoid. Outliers (in Surveillant and Primed) were removed from further analysis

Since the elbow plot did not clearly indicate the optimal number of clusters in our data (see Fig. 2B), we further assessed k using “gap statistics”. This technique evaluates the optimal number of clusters by comparing the within-cluster dispersion (how tightly the clusters are packed) of the obtained clustering results to that of a randomly generated reference dataset. The highest gap value signifies a more distinct clustering structure, assisting in the identification of the optimal number of clusters (k) [62]. The higher the gap statistic, the more the clusters differ from a random structure, representing real patterns in the data.

After determining the optimal number of clusters, we employed uniform manifold approximation and projection (UMAP) to visualise the high-dimensional data in a more manageable, lower-dimensional space. UMAP is a sophisticated, non-linear dimensionality reduction technique specifically designed to preserve both local and global structures within the dataset. It ensures that points close to one another in the high-dimensional space remain nearby in the projected lower dimensions while also maintaining the overarching organisation of the data. Unlike traditional linear methods such as principal component analysis (PCA), which may oversimplify complex relationships, UMAP proficiently captures intricate, non-linear patterns, making it particularly valuable for clustering and exploratory data analysis. By projecting the data into two dimensions, UMAP allows for a more discernible representation of cluster separations and reveals the underlying patterns that may otherwise remain hidden in higher dimensions [33]. Unlike PCA, the axes in UMAP do not correspond to any biological variable. They represent a compressed version of the original space, where proximity indicates similarity.

To visualise the high-dimensional dataset and identify potential clustering patterns among cell populations, the UMAP analysis was performed based on the scaled and filtered feature set. This analysis was conducted using the** “umap”** package in R. The Euclidean distance metric was applied to measure the similarity between data points, and the nearest neighbour search was optimised using the Fast Nearest Neighbor “fnn” method to improve computational efficiency. The UMAP parameters were set as “**n_neighbors = 7”** to balance local and global structure and “**min_dist = 0.001”** to control the spread of clusters. All data were pre-processed to ensure consistent scaling across features. Finally, the resulting UMAP embeddings were examined to assess the distribution and relationships among different cell groups.

Further, to determine the clustering of the cells, we employed the partitioning around medoids (PAM) clustering method, a medoids-based clustering algorithm that enhances the robustness of the clustering process. Unlike traditional k-means methods that use the mean value of data points to define cluster centres, PAM selects actual data points from the dataset, known as medoids. This key distinction makes PAM less sensitive to outliers, as the medoid represents a data point that minimises the distance to all other points in its cluster. In this study, we used Euclidean distance as the similarity measure to determine the proximity between data points, ensuring that cells with similar morphological features were grouped together. Consequently, PAM is particularly effective for handling non-spherical clusters where the distribution of data does not conform to typical geometric shapes [40, 41].

### Microglial state classification

We are aware of the difficulty in the nomenclature and classification of microglia [39] as their states exist along a dynamic continuum rather than within rigidly defined categories. For reporting purposes, we adopt a classification based on three primary states: surveillant, primed, and activated. These functional states have previously been reported to be associated with specific morphological features (phenotypes). These phenotypes were identified in an automated fashion by our cluster analysis approach (see above).

(A) *Surveillant* microglia correspond to the “ramified” morphology [39], with small cell bodies and long, dynamic processes. Thus, the cell volume is low in this state, but the cell territorial volume is higher due to the presence of thin and long processes. These processes contain a higher number of branches and branch points. These microglia are thought to represent the “homeostatic” state, in which they are actively engaged in continuous surveillance of the CNS microenvironment. In this baseline physiological condition, they are critical in maintaining tissue homeostasis and synaptic integrity.
(B) *Primed* microglia correspond to a “rod-like” morphology [6] with elongated cell bodies and retracted processes. In our study, the cell volume can be higher or lower in this state, but the cell territorial volume is lower than in surveillant microglia. The number of branch points and branches is also reduced compared to the surveillant cells. These microglia are thought to represent an “intermediate” state, in which they have undergone a subtle functional activation in response to previous stimuli or a change in the environment but are not fully activated or inflammatory.
(C) *Activated* microglia are characterised by their “amoeboid” morphology [39, 52] with cell body enlargement and process retraction. In our study, the cell volume significantly increases in this state, exhibiting few or no processes. These microglia are thought to represent a “phagocytic” state, in which they are activated in response to acute or chronic insults.

### Data analysis

Statistical analyses were performed using R Studio v4.3.0 (2023.9.1.494). Normal distribution of the data was tested with the Kolmogorov–Smirnov test. The data fit the rules of parametric analysis. The proportion of microglial cells were calculated per rat and averaged across animals, giving equal weight to each rat. Finally, the values were normalized so that the total for each state summed to 100% within each group. All comparisons were performed using the independent *t*-test, as only two groups were compared simultaneously. Two-way ANOVA was performed to determine the interaction between sex and microglia states. Statistical significance was considered with an alpha level of 0.05 or lower (*p* < 0.05, two-tailed). In addition, we report effect sizes calculated between groups using Cohen’s *d* as the mean difference divided by the pooled SD [28]. Cohen’s *d* between 0.2 and 0.5 indicated a small effect size, Cohen’s *d* between 0.5 and 0.8 indicated a medium effect size, and Cohen’s *d* > 0.8 indicated a large effect size. Reporting of effect size will permit power calculations for future studies.

## Results

### Microglial state classification

A total of 3573 cells were segmented from three-dimensional images of the dorsal horn ipsilateral to the injection sites in the multifidus muscle. After removing the cells with any missing feature values (see methods Sect. 2.6), 3516 cells were used for subsequent analyses. The features were scaled to ensure consistent weighting using z-score normalization.

Before identifying clusters of cells based on morphological features (phenotypes), we looked for redundancies in the 8-dimensional feature space by cross-correlation (Fig. 2A). The number of branch points and endpoints were highly correlated (*r* = 0.97). The number of endpoints as a feature was removed from further analysis due to its higher absolute correlation with the remaining features (mean absolute correlation of number of endpoints, *r* = 0.5; mean absolute correlation of number of branchpoints, *r* = 0.4).

Morphological classification of microglia states was done using distributive cluster analyses. First, an elbow plot was used in an attempt to estimate the optimal number of clusters (*k*) (Fig. 2B). However, due to a curved elbow and no sharp bend, the transition from deep to shallow resulted in an optimal number of clusters between two and four. As an alternative, we used the gap statistic; the highest Gap statistics value was recorded for *k* = 3 (Fig. 2C). Hence, the optimal number of clusters for the dataset was identified as three.

The scaled 7-dimensional feature space was reduced for dimensionality using UMAP, followed by PAM clustering to visualise the cells in three distinct clusters (Fig. 2D). Morphological microglia phenotypes were assigned functional states (see Methods 2.8). For the surveillant state, 14 of 1592 cells were outliers. For the primed state, 5 of 1647 cells were outliers. These outliers were eliminated, leaving behind a total of 3473 microglial cells for subsequent analyses.

Table 2 illustrates the mean features of the resulting three microglia phenotypes. The transition from surveillant to primed state was mostly characterised by decreases in cell territorial volume (Cohen’s *d* = 1.54, *p* < 0.0001) and cell volume (Cohen’s *d* = 1.33, *p* < 0.0001). In contrast, transitioning from the primed to the activated state of microglia was associated with decreases in average branch length (Cohen’s *d* = 2.63, *p* < 0.0001) and maximum branch length (Cohen’s *d* = 2.56, *p* < 0.0001).

**Table 2:** Cluster characteristics

Features	Surveillant	§d	Primed	^d	Activated
No. of cells	1548		1642		277
Cell territorial vol (1000 µm^3^)	121.5 ± 79.6	**1.54**	32.5 ± 22.4	0.94	12.5 ± 9.9
Cell volume (1000 µm^3^)	17.6 ± 10.7	**1.33**	7.1 ± 3.8	0.62	4.6 ± 2.9
Ramification index	7.4 ± 3.2	0.97	4.7 ± 2.1	1.03	2.6 ± 1.1
Number of branch points	16.1 ± 13.1	1.15	5.2 ± 2.8	1.97	0.0 ± 0.0
Average branch length (µm)	104.1 ± 38.8	1.37	59.7 ± 24.5	**2.63**	0.0 ± 0.0
Max branch length (µm)	222.5 ± 130.9	1.23	103.7 ± 43.7	**2.56**	0.0 ± 0.0
Min branch length (µm)	21.4 ± 11.9	0.17	23.9 ± 16.9	1.53	0.0 ± 0.0

The table represents the different characteristics based on which the clusters were classified. The number of cells represents each cluster’s total number of cells. The columns, as mentioned, represent the median of the features in each cluster (surveillant, primed, and activated), used to assign the cell morphology type to each cluster. The ramification index is the ratio between the cell territorial and cell volumes. The section sign “§” represents Cohen’s *d* of the features difference while transitioning from surveillant to primed state of microglia. The circumflex accent “^” represents Cohen’s *d* on the state of the features difference while transitioning from primed to activated state of microglia. The magnitude of the effect sizes were determined by ###: *d* > 0.8 refers to a large effect, ##: 0.8 > *d* > 0.5 refers to a medium effect, #: 0.5 > *d* > 0.2 small effect

### Sex differences in microglial states

**Fig. 3:**
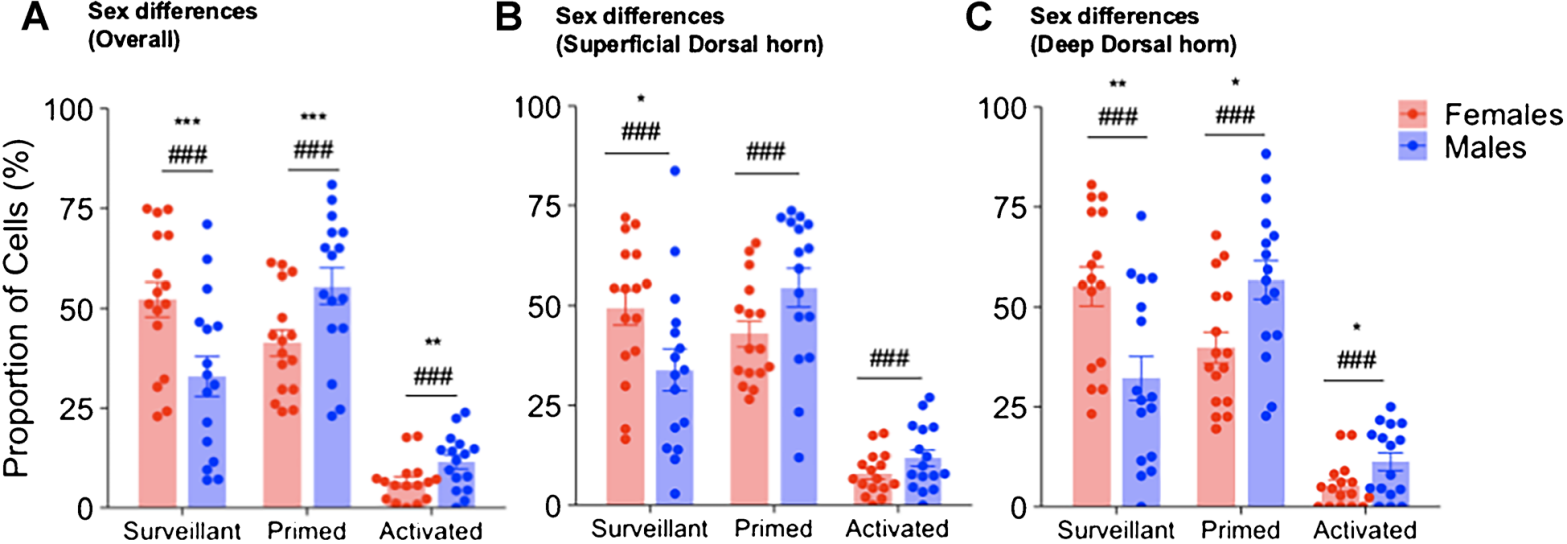
Sex differences in the activated microglial states. Sex difference analysis of the microglial states across all four treatment groups (in Methods Sect. 2.4). All groups were activated before sacrifice. **A** Sex difference in the proportion of microglial cells (%) in each state across all four regions of interest (ROIs). **B** Sex difference in the proportion of microglial cells in each state within the superficial dorsal horn region (ROI1 + ROI2) of the spinal cord. **C** Sex differences in the proportion of the microglia cells within the deep dorsal horn region (ROI3 + ROI4) of the spinal cord. Data are reported as mean ± SEM. Significance according to independent *t*-test: *p < 0.05, **p < 0.01, ***p < 0.001. Effect sizes according to Cohen’s *d*: ### *d* > 0.8 refers to a large effect, ## 0.8 > *d* > 0.5 refers to a medium effect, # 0.5 > *d* > 0.2 small effect. *n* (males) = 16 and *n* (females) = 16

All four treatment groups included the potential for microglia activation by adolescent stress and/or by intramuscular NGF injection. We evaluated overall sex differences in microglia phenotypes by combining data from all four treatment groups. Regarding location, we examined microglia states across all four regions of interest (ROIs) and separately in the superficial and deep dorsal horn regions. The analysis across all four ROIs (Fig. 3A) revealed that females exhibited significantly more surveillant microglia (*p* < 0.0001, Cohen’s *d* = 1.92), whereas males had significantly higher levels of primed microglia (*p* < 0.0001, Cohen’s *d* = 1.62) and activated microglia (*p* < 0.01, Cohen’s *d* = 1.96). The two-way ANOVA between sex and microglia state changes showed a highly significant but medium-effect interaction (*p* < 0.001, Cohen’s *d* = 0.59). Similar sex differences were observed when analysing ROIs in superficial (Fig. 3B) and deep dorsal horn (Fig. 3C) separately. The interaction between sex and microglia state in the superficial dorsal horn was significant with a medium effect size (*p* < 0.001, Cohen’s *d* = 0.55). The interaction between sex and microglia state in the deep dorsal horn region had a significant medium-sized effect (*p* < 0.001, Cohen’s *d* = 0.75). Effect sizes are summarised in Table 3. This pattern is supposed to indicate a smaller degree of activation in females than in males, with fewer microglia reaching the morphological equivalent of full activation throughout the dorsal horn. In the following analyses, data were pooled across all ROIs.

**Table 3:** Sex differences in microglia states

**Microglia state**	**Groups**	**No. of cells (males)**	**No. of cells (females)**	***p***		***d***	
Surveillant	Overall	457	1091	0.0000	***	1.92	###
Primed	Overall	820	822	0.0000	***	1.62	###
Activated	Overall	154	123	0.0014	**	1.96	###
Surveillant	Superficial DH	264	555	0.0297	*	1.98	###
Primed	Superficial DH	415	466	0.0542		1.45	###
Activated	Superficial DH	77	80	0.1140		2.14	###
Surveillant	Deep DH	193	536	0.0038	**	1.80	###
Primed	Deep DH	405	356	0.0101	*	1.59	###
Activated	Deep DH	77	43	0.0314	*	1.56	###

The table represents statistical sex differences in the microglia states. The Overall group represents the sex differences in the distribution of the microglia states pooled across treatment groups. The superficial DH represents sex differences in the distribution of the microglia states in the superficial dorsal horn region of the spinal cord pooled across treatment groups. The deep DH represents the sex differences in the distribution of the microglia states in the deep dorsal horn region of the spinal cord, pooled across treatment groups. The *p*-value (*p*) is calculated using the independent *t*-test (*n* = 16). The number of cells in the male and female columns represents the number of microglia in each state included in the analysis. The significance is denoted by: *p < 0.05, **p < 0.01, ***p < 0.001. Cohen’s *d* (*d*) is calculated by differences in the mean values divided by pooled SD. The magnitude of the effect sizes were determined by ###: *d* > 0.8 refers to a large effect, ##: 0.8 > *d* > 0.5 refers to a medium effect, #: 0.5 > *d* > 0.2 small effect

### Differences between the treatment groups

**Fig. 4:**
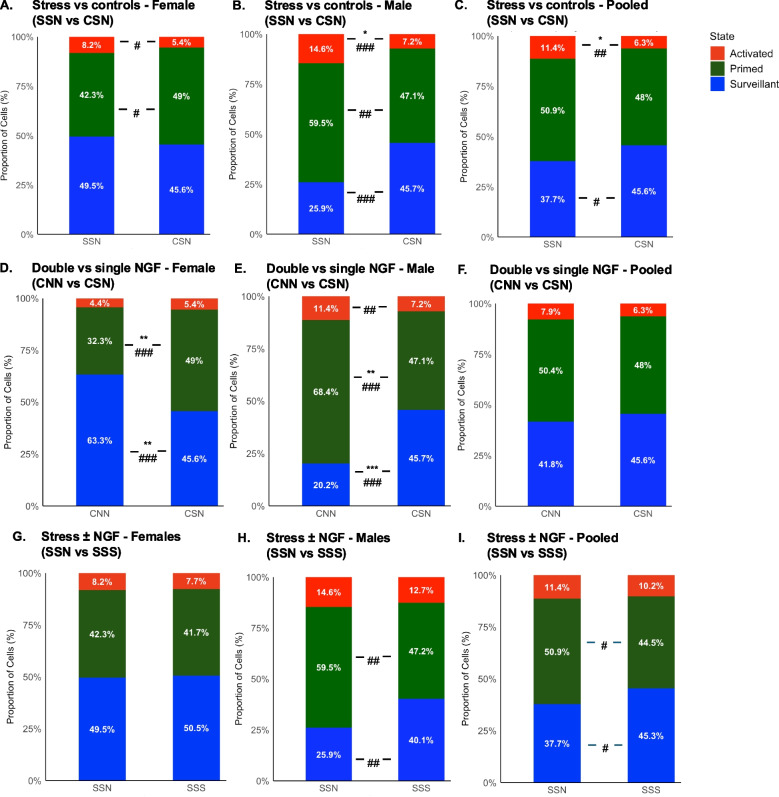
Different priming effects on microglia states. The differences between different treatment groups. **A** Priming by stress: The difference between the stress vs controls with one single NGF in adulthood in females. **B** SSN vs CSN in males. **C** SSN vs CSN for overall. **D** Priming by NGF: The difference between controls with two NGF vs single NGF injection in females. **E** CNN vs CSN in males. **F** CNN vs CSN for overall. **G** Additive effect of Stress ± NGF: The differences between the stressed animals with NGF vs Saline as second injections in females. **H** SSN vs SSS in males. **I** SSN vs SSS for overall. Data is reported as the mean per animal of the proportion of microglia (%) (n = 4). Significance according to independent t-test: *p < 0.05, **p < 0.01, ***p < 0.001. Effect sizes according to Cohen’s d: ###d > 0.8 refers to a large effect, ##0.8 < d > 0.5 refers to a medium effect, #0.5 > d > 0.02 small effect. CSN: Control+Saline+NGF; SSN: Stress+Saline+NGF; SSS: Stress+Saline+Saline; CNN: Controls+NGF+NGF

In order to maximise sensitivity to detect differences between treatment groups, data from both sexes were analysed separately as well as pooled together. Priming by adolescent stress for microglia activation in adulthood was assessed by comparing stress vs control groups with saline injection on PD 85 and a single NGF injection on PD 90 (SSN: Stress + Saline + NGF vs CSN: Control + Saline + NGF). Priming by stress in females only showed non-significant small effect size reduction in the proportion of primed state (*p* > 0.05, Cohen’s *d* = 0.42) and increase in activated state (*p* > 0.05, Cohen’s *d* = 0.43; Fig. 4A). In males, priming by stress demonstrated significant medium effect size increases in the proportions of activated cells (*p* < 0.05, Cohen’s *d* = 0.82) and increase in primed cells (*p* < 0.05, Cohen’s *d* = 0.64) (Fig. 4B). A slight effect size decrease in the surveillant (*p* > 0.05, Cohen’s *d* = 0.37) microglia state and a medium effect size and significant increase in activated cells in Stress + Saline + NGF were reported in the pooled data. The proportion of primed microglia remained relatively constant (Fig. 4C, Table 4). Priming by stress caused microglia to transition from a surveillant to an activated state. This change was found to be more noticeable in males than in females, but it remained significant when data were pooled across both sexes.

**Table 4:** Differences between microglia states within sexes and in between the injection groups

		**Females**					**Males**				**Pooled**			
**Condition**	**Microglia state**	**Groups (No. of cells)**			***p***	***d***	**Groups (No. of cells)**		***p***	***d***	**Groups (No. of cells)**		***p***	***d***
		SSN	CSN				SSN	CSN			SSN	CSN		
Priming by stress: SSN vs. CSN	Surveillant	250	254	0.5700		0.21	090	172	0.1409	0.97^###^	340	426	0.1410	0.37^#^
	Primed	196	242	0.2460		0.42^#^	226	160	0.5414	0.64^##^	422	402	0.5410	0.15
	Activated	34	28	0.2360		0.43^#^	41	25	0.0145*	0.82^###^	75	53	0.0146*	0.63^##^
		CNN	CSN				CNN	CSN			CNN	CSN		
Priming by NGF: CNN vs. CSN	Surveillant	178	254	0.0084**		0.99^###^	092	172	0.0009***	1.32^###^	270	426	0.5190	0.16
	Primed	337	242	0.0037**		1.13^###^	290	160	0.0017**	1.23^###^	627	402	0.6491	0.14
	Activated	23	28	0.6060		0.18	53	25	0.1440	0.53^##^	76	53	0.4030	0.21^#^
		SSN	SSS				SSN	SSS			SSN	SSS		
Additive effects of stress ± NGF: SSN vs. SSS	Surveillant	250	250	0.8800		0.05	90	103	0.0840	0.64^##^	340	353	0.1810	0.34^#^
	Primed	196	196	0.9220		0.03	226	144	0.0904	0.62^##^	422	340	0.1690	0.35^#^
	Activated	34	34	0.8340		0.07	41	35	0.6540	0.16	75	69	0.6290	0.12

The table represents the statistical differences in microglial states across treatment groups in females, males, and a pooled (across sexes) group. SSN vs. CSN compares the differences between the priming effects of stress and the control group. CNN vs. CSN compares the priming effect of NGF with that of the control group. SSN vs. SSS compares the priming effect of stress with single and no NGF injection. Groups (no. of cells) represent the involved groups with the number of cells used for calculations in each case. The *p*-value is calculated using the independent *t*-test. The significance is denoted by: *p < 0.05, **p < 0.01, ***p < 0.001. Cohen’s *d* is calculated by differences in the mean values divided by the pooled SD. The magnitude of the effect sizes was determined by ###: *d* > 0.8 refers to a large effect, ##: 0.8 > *d* > 0.5 refers to a medium effect, #: 0.5 > *d* > 0.2 refers to a small effect. SSN: Stress + Saline + NGF; SSS: Stress + Saline + Saline; CSN: Control + Saline + NGF; CNN: Control + NGF + NGF. For each group tested, *n* = 4

Microglia priming by a mild nociceptive input (first NGF injection) was assessed by comparing non-stressed control groups that received either two NGFs or a single NGF preceded by saline (CNN: Control + NGF + NGF vs. CSN: Control + Saline + NGF). Females had a significantly increased proportion of surveillant cells in the two NGF group (CNN: *p* < 0.01, Cohen’s *d* = 0.99) and a significant and large effect increase in the proportion of primed cells in the single NGF group (CSN: *p* < 0.01, Cohen’s *d* = 1.13; Fig. 4D). Whereas in the two NGF group in males (CNN), there was a significant large effect size increase in the proportion of primed cells (*p* < 0.01, Cohen’s *d* = 1.23) and a non-significant but medium effect size increase in the proportion of activated cells (*p* < 0.05, Cohen’s *d* = 0.53). The single NGF group in males (CSN) showed a significant increase in the proportion of surveillant cells (*p* < 0.001, Cohen’s *d* = 1.32; Fig. 4E). The results provide no evidence for a priming effect on the behaviour of females of the first conditioning NGF injection on the second test NGF injection, as females possess more surveillant than activated or primed microglia (behaviour data in Singhal et. al., 2025). In alignment with a previous study in male Sprague–Dawley rats [69], we found evidence for microglial priming of the first conditioning NGF injection on the second test NGF injection in males. However, the opposite effects across sexes got cancelled out in the pooled differences observed in all three states of microglia (Fig. 4F, Table 4). Therefore, the comparison between two vs. one NGF injections in non-stressed animals indicated that mild nociceptive priming activated microglia only in males. This effect was not statistically significant when pooled across both sexes.

A potential additive effect between microglia priming in adolescence and acute microglia activation by NGF was assessed by comparing stressed animals with single vs no NGF injection (SSN: Stress + Saline + NGF vs SSS: Stress + Saline + Saline). No changes were reported in SSN vs SSS groups in females in either of the microglia states (Fig. 4G). However, in males, a non-significant medium effect size increase in the proportion of primed cells was reported within the SSN group (*p* > 0.05, Cohen’s *d* = 0.62). The male SSS group showed a non-significant medium effect size increase in the proportion of surveillant cells (*p* > 0.05, Cohen’s *d* = 0.64; Fig. 4I). When pooled across sexes, we observed a non-significant small effect size increase in the proportion of primed microglia (*p* > 0.05, Cohen’s *d* = 0.35) and a non-significant small effect size decrease in the proportion of surveillant microglia (*p* > 0.05, Cohen’s *d* = 0.34) (Fig. 4J, Table 4). The additivity of priming by stress in adolescence and challenge by NGF injection in adulthood was observed as a shift towards a primed state only in males, not in females.

The detailed number of cells studied in each microglial state, *p*-values, and Cohen’s *d* for males, females, and pooled data are reported in Table 4.

## Discussion

Analysing microglial morphology is challenging due to its complexity and dynamic nature [39]. We established an unbiased semi-automated approach to stratify microglial cells based on seven 3-dimensional morphological features by unsupervised machine learning (clustering): surveillant, primed, and activated. Overall, microglial cells included in this study showed a significantly higher proportion of surveillant microglia in females, while males showed significantly higher proportions of primed and activated microglia. The distribution of microglia across the three phenotypes did not differ between the superficial and deep dorsal horn in both sexes. When comparing microglia state distribution after a single NGF injection with and without priming by restraint stress in early life, males showed a marked shift towards the activated and primed state, while females only showed a small, insignificant increase in activated microglia. A similar shift in microglia state distribution was observed in males but not females after two NGF injections as compared to a single NGF injection. A single NGF injection after restraint stress in early life had an additive effect in males, but not in females. As the effects of early-life stress from PD 21 to PD 32 are still evident in adulthood at PD 91, we propose our model is a valuable preclinical tool to study neurobiological changes induced by adverse childhood experiences (ACEs). We show that early-life stress influences microglial states in the long term, suggesting microglia-dependent latent sensitisation in the spinal cord dorsal horn as a pathophysiological link between ACEs and cpLBP. Similar sex differences had been reported for neuropathic pain models.

### 3D classification of microglia morphology

Microglia state classification has been reported on different levels of complexity (morphology, transcriptomics, metabolomics, proteomics, functional) rather than the dichotomic distinction between a resting M1 and an activated M2 state [39]. In this study, we employed an automated MATLAB-based method for 3D segmentation of the microglial cells [68] to eliminate investigator bias. Manual analysis is prone to observer bias and unsuitable for high-throughput studies. With the help of this tool, we were able to extract eight features for 3516 cells. Using the automated clustering analyses, we grouped the cells with similar morphological features into three distinct phenotypes assumed to correspond to microglia functions [39]. 3D classification of microglia is generally more helpful and effective than 2D approaches because it permits unequivocal assignment of branches to their parent soma [5]. By capturing the entire cell in its native tissue context, 3D imaging also avoids partial visualization errors. It allows for precise measurements of features such as soma volume, branching complexity, and spatial clustering. Our data on male animals are a refined re-analysis of a study by Singaravelu and colleagues [53]. While they reported only minor microglial activation after restraint stress, we now found a significant increase over controls. By employing automated 3D analysis instead of manual 2D analysis, we included a significantly higher number of cells, thereby increasing sensitivity. Moreover, the automated procedure provides for higher reliability.

### Microglia state classification

Microglia are the resident immune cells in the central nervous system and display various functional states in response to pathological conditions, such as stress-induced cpLBP. As microglia morphology and their functional states are interconnected, we clustered the cells into three distinct phenotypes based on their morphological features: surveillant, primed, and activated. In their homeostatic surveillant state under normal physiological conditions, microglia are highly ramified for active immune monitoring and the preservation of neuronal homeostasis, which results in a high cell territorial volume and a high number of branchpoints [13, 26, 38, 39, 50]. Primed microglia exist in an intermediate state, sensitised by prolonged or repeated stress, in which their cell territorial volume and cell volume is markedly decreased compared to surveillant microglia (Table 2), but they do not produce high levels of proinflammatory mediators [37, 64, 66]. As their fully reactive phenotype, which contributes to neuroinflammation, in the activated state, microglia morphology evolves towards an amoeboid shape reflected in a distinct decrease in average and maximum branch length (Table 2). Activated microglia release cytokines, engage in phagocytosis, and interact with peripheral immune cells in response to stressful events or insults [27, 50, 65]. Even though microglia morphology describes a continuum that is correlated but not strictly representative of function, this classification balances biological relevance, analytical feasibility, and potential for translation into clinical applications [50].

### Microglia in latent sensitisation

Microglia are thought to play a crucial role in latent sensitisation, a state of heightened pain sensitivity that remains asymptomatic until triggered by external stimuli (e.g. nociceptive input through an NGF injection) [69]. Activated microglia release pro-inflammatory cytokines such as TNF-α and IL-1β via pathways like fractalkine signalling and P2X7R/NLRP3 inflammasome activation, enhancing synaptic excitability in the spinal dorsal horn and facilitating spinal sensitisation [63]. Fractalkine (CX3CL1) released from primary afferents and dorsal horn neurons amplifies nociceptive signalling via CX3CR1 receptors on microglia [15, 29] In models of NGF-induced myofascial pain, blocking microglial activation prevents spinal sensitisation and disrupting fractalkine signalling reduces hypersensitivity [51] emphasising the importance of the CX3CL1-CX3CR1 axis [23, 29, 69].

We found that early-life physical stress primed the microglia, resulting in a higher proportion of activated microglia upon NGF injection in adulthood than in unstressed controls. This priming effect was more pronounced in males than in females. The exact pathophysiological mechanisms through which early-life stress induces long-term latent sensitisation are unknown. The involvement of fractalkine signalling is probable, as it is involved in other stress-related phenotypes: inhibiting fractalkine signalling prevented changes in microglia function and depression-like behaviours in CX3CR1 knockout mice following stress [34]. Human studies have also shown a positive relationship between fractalkine signalling and major depressive disorder [11]. Our data suggest that the predisposing effects of early-life stress for the development of cpLBP later in life are mediated through latent sensitisation-like modulation of microglia.

### Sex differences in microglial activation

Sex differences in microglial activation, resulting from early life stressors, are crucial in shaping neurological and pain-related outcomes in adulthood. In this study, changes in microglia numbers were not included in the analysis, as no differences in microglia quantity have been previously reported between sexes when measured using Iba-1 immunostaining, flow cytometry, or gene expression [36].

Sex differences of acute pain-producing actions of intramuscular NGF injections have been studied in humans (masseter and tibialis anterior muscles) and rodents (multifidus muscle). Since injections in rodents are done under anesthesia, data on pain upon injection is only available for humans: women had same peak pain as men [2], had higher peak pain than men [4] or lower peak pain [49]. Both sexes exhibited mechanical hyperalgesia later, which was more pronounced in women than men in two studies with masseter injections; the one study with similar hyperalgesia across sexes [4] used tibialis injections that were more painful in men, so the conditioning stimulus may have been less effective in women. The overall picture in humans suggests a similar painfulness of the NGF injections but stronger sensitisation in women than men. Sensitivity to painful mechanical stimulation is higher in females for both humans and rats. Both sexes in rodents exhibit hypersensitivity to mechanical stimuli after NGF injection (three studies in Sprague–Dawley rats by Reed and colleagues [44, 45, 57]). Our study in Wistar-Han rats also demonstrated stronger sensitisation of females than males by a first NGF injection [55] but females habituated to the second injection while males sensitised.

Our current findings demonstrate a clear sex-specific divergence in microglial responses to early-life stress and NGF-induced pain in adulthood and their combination. Females not exposed to early-life stress showed a higher proportion of surveillant microglia after two rather than a single NGF injection. However, after early-life stress, the proportion of activated microglia was higher than in unstressed females. In contrast, males exhibited more primed and activated microglia upon two NGF injections rather than one, as well as higher NGF-induced microglia priming and activation in animals subjected to early-life stress, suggesting a microglia-dependent interplay between early-life stress and NGF susceptibility in adulthood. This reveals a novel pattern, in which microglia in males are responsive to both early-life stress and nociceptive insults in adulthood, i.e. latent sensitisation is induced by both stimuli and then escalated to manifest sensitisation upon a second hit in adulthood. Microglia in females, however, are responsive to early-life stress, but no additional changes were induced by the application of NGF in adulthood.

Studies in nerve injury models of peripheral neuropathic pain have shown that sex differences in microglia activation shape distinct neuroimmune mechanisms in pain processing [67]. Males rely on microglial pathways like P2X4 receptor upregulation [30], p38 MAPK signalling [59], and the brain-derived neurotrophic factor (BDNF)-dependent neuronal inhibition pathways [7]. Microglia in males are activated, leading to the upregulation of P2X4 receptors. The stimulation of these receptors triggers the release of BDNF through a p38 signalling pathway. BDNF binds to trkB receptors on neurons, resulting in reduced *KCC2* expression, which in turn decreases inhibition and increases pain signalling. It has been reported that the deletion of BDNF from microglia prevented SNI-induced pain hypersensitivity only in males [56, 70]. However, advances in single-cell RNA sequencing revealed that spinal microglia do not exhibit *Bdnf* gene expression [17]. Therefore, discussing the role of microglial BDNF in dorsal horn neuron hypersensitivity remains challenging. However, in females, microglial BDNF is not the primary source [30]. In contrast, females depend more on adaptive immune cells such as T lymphocytes, hormonal influences like estrogen, and GLP-1 receptor modulation, although the underlying molecular mechanisms are still unclear [30]. However, some studies have presented conflicting evidence, indicating that P2X4 receptor inhibition also impacts female rats [19, 24, 31]. Notably, in the absence of adaptive immune cells, neuroimmune interactions in females shift to rely on the microglial pathway, similar to males [30, 56]. Our data suggest that these sex-specific differences also come into play in an animal model of musculoskeletal pain, which has been less explored than the extensively studied models of neuropathic pain.

In our study in a combined stress plus mild nociceptive input model of chronic musculoskeletal pain, most microglia were found to be in a primed state in both sexes. Surveillant microglia were more prevalent in females than males, possibly because in females, priming or sensitisation of dorsal horn neurons in the spinal cord is primarily based on T-cell-dependent mechanisms. Hence, the maintenance of the surveillant microglia, along with the previous evidence of T-cell involvement in pain, suggests an alternative, slower-developing immune pathway that supports latent sensitisation as well. Whereas in males, mainly microglia-mediated mechanisms contribute to neuronal sensitisation. Microglia priming by early life stressors can significantly influence the modulation of pain pathways, resulting in variations in pain sensitivity between males and females [14]. Hence, males might be more affected by manifest sensitisation through NGF after both NGF- and stress-induced latent sensitisation, as they are more dependent on microglia-mediated mechanisms to regulate excitability of spinal cord nociceptive pathways.

### Strengths and limitations

A major strength of our study is that we used a 3D automated machine-learning tool to classify microglia based on their morphological features, thereby minimizing the investigator bias that is common in manual analysis. By clustering cells into three states, we achieved a balance between biological relevance and analytical feasibility. For limitations, to abide by the 3R principles of animal use, we reused the raw 3D acquired confocal images for the male cohort from the previous study, where animals were handled by a different experimenter, limiting comparability [53]. We also lack a negative control (Control + Saline + Saline), as previous electrophysiological studies [22, 69] reported no difference between two saline injections and latent sensitisation by one NGF injection.

### Summary and conclusions

This study examined changes in microglial phenotypes in the L2 dorsal horn of the spinal cord using a 3D automated phenotyping method. Across all groups, males had more activated microglia, whereas females showed more surveillant cells. Priming by stress caused microglia to shift from surveillant to activated state. Thus priming by stress during adolescence had a similar sensitising effect on microglia responsiveness to mild nociceptive input in adulthood as previously found for preceding nociceptive input. These findings suggest a critical role for microglia in the pathophysiology of cpLBP, which is known clinically to develop after mild injuries. Multiple such injuries appear to be necessary for manifest cpLBP. Moreover, stress was confirmed to play a critical role in priming microglia and predisposing for cpLBP. This change was more noticeable in males than females, but remained significant when considering both sexes together. We also found additive effects of priming by stress and challenge by NGF as a shift towards the primed state in males, not females. These findings correlated with behavioural observations [55] and suggested that males are more prone to manifest sensitisation. Sex differences previously shown for neuropathic pain, were found to be also relevant in musculoskeletal pain condition which are more prevalent clinically.

## Data Availability

The raw confocal microscopy images used in this study were acquired and analysed by the authors as part of the experimental workflow. These images were used for quantitative and qualitative data generation. The raw image data are not publicly available due to file size limitations but are available from the corresponsing author upon reasonable request. However, the R-based analysis pipeline used for data processing is freely available at: https://github.com/DeepikaSinghal14/Microglia-analysis.git.
